# Sodium Butyrate-Modulated Mitochondrial Function in High-Insulin Induced HepG2 Cell Dysfunction

**DOI:** 10.1155/2020/1904609

**Published:** 2020-07-16

**Authors:** Tingting Zhao, Junling Gu, Huixia Zhang, Zhe Wang, Wenqian Zhang, Yonghua Zhao, Ying Zheng, Wei Zhang, Hua Zhou, Guilin Zhang, Qingmin Sun, Enchao Zhou, Zhilong Liu, Youhua Xu

**Affiliations:** ^1^Faculty of Chinese Medicine, State Key Laboratory of Quality Research in Chinese Medicine, Macau University of Science and Technology, Taipa, Macao, China; ^2^Institute of Chinese Medical Sciences, State Key Laboratory of Quality Research in Chinese Medicine, University of Macau, Taipa, Macao, China; ^3^Department of Endocrinology, Zhuhai Hospital of Integrated Traditional Chinese and Western Medicine, Zhuhai, China; ^4^Jiangsu Province Hospital of Traditional Chinese Medicine, Affiliated Hospital of Nanjing University of Chinese Medicine, Hanzhong Road, Nanjing, China

## Abstract

The liver plays a pivotal role in maintaining euglycemia. Biogenesis and function of mitochondria within hepatocytes are often the first to be damaged in a diabetic population, and restoring its function is recently believed to be a promising strategy on inhibiting the progression of diabetes. Previously, we demonstrated that the gut microbiota metabolite butyrate could reduce hyperglycemia and modulate the metabolism of glycogen in both db/db mice and HepG2 cells. To further explore the mechanism of butyrate in controlling energy metabolism, we investigated its influence and underlying mechanism on the biogenesis and function of mitochondria within high insulin-induced hepatocytes in this study. We found that butyrate significantly modulated the expression of 54 genes participating in mitochondrial energy metabolism by a PCR array kit, both the content of mitochondrial DNA and production of ATP were enhanced, expressions of histone deacetylases 3 and 4 were inhibited, beta-oxidation of fatty acids was increased, and oxidative stress damage was ameliorated at the same time. A mechanism study showed that expression of GPR43 and its downstream protein beta-arrestin2 was increased on butyrate administration and that activation of Akt was inhibited, while the AMPK-PGC-1alpha signaling pathway and expression of p-GSK3 were enhanced. In conclusion, we found in the present study that butyrate could significantly promote biogenesis and function of mitochondria under high insulin circumstances, and the GPR43-*β*-arrestin2-AMPK-PGC1-alpha signaling pathway contributed to these effects. Our present findings will bring new insight on the pivotal role of metabolites from microbiota on maintaining euglycemia in diabetic population.

## 1. Introduction

Type 2 diabetes (T2D) has become a major threat to health worldwide. It is estimated that the diabetic population will rise to 600 million people within the next 20 years, accounting for about 10% of the world population. The liver plays a pivotal role in maintaining euglycemia; unfortunately, as high as 19% of cases with type 2 diabetes are reported being accompanied with liver dysfunction [1].

The liver is one of the main target organs for insulin. By modulating glycogenesis or glucose oxidation within hepatocytes, blood glucose is maintained in a relatively stable state. However, a very high level of insulin, or the so-called insulin resistance (IR), will significantly destroy the capacity of the liver in this aspect, and the function and biogenesis of mitochondria are often the first to be damaged [2, 3]. In this sense, restoring the function of mitochondria is pivotal to inhibit the progression of T2D.

With the understanding of the important role of gut microbiota in disease development, interests have focused on exploring the mechanism of a potential target for controlling T2D. In 2012, Qin and colleagues firstly demonstrated that butyrate-producing bacteria were significantly reduced in a T2D population [4]. Thereafter, studies suggested the potential role of butyrate supplementation on modulating diabetes [5, 6]. Previously, we demonstrated in db/db mice that oral administration with sodium butyrate (NaB) significantly reduced HbA1c and diabetic inflammation [7]; more importantly, hypertrophy and steatosis of hepatocytes in db/db mice were significantly reversed by NaB, accompanied with enhancement of glycogen metabolism [8]. To further investigate the potential role of NaB on mitochondria, we carried out a series of experiments to observe both the biogenesis and function of mitochondria under high insulin circumstances in this study; the underlying mechanism was also explored. Our present study may bring new insight on understanding the pivotal role of metabolites from microbiota in controlling energy metabolism.

## 2. Materials and Methods

### 2.1. Materials

Sodium butyrate (NaB) was provided by Meilun Biological Technology (Dalian, China). Antibodies or agents for GAPDH (sc-47724), GPR43 (sc-32906), *β*-arrestin2 (sc-13140), Akt (sc-514032), p-Akt (sc-8312), GSK3*α*/*β* (sc-7291), p-GSK3*α*/*β* (sc-81496), GPR43-siRNA (sc-77339), control siRNA-A (sc-37007), DCFH (sc-359840), and JC-1 iodide(sc-364116) were purchased from Santa Cruz (Dallas, TX); AMPK (5832s) and p-AMPK (2531s) were purchased from Cell Signaling Technology (Danvers, MA); PGC1-alpha (ab54481) was purchased from Abcam (Cambridge, UK); and insulin receptor (bs-0681R) was purchased from BIOSS (Greater Boston, New England). The QIAamp® DNA Micro Kit (56304) and RT^2^ Profiler™ PCR Array Human Mitochondrial Energy Metabolism (330231) were from QIAGEN (Hilden, Germany); LongAmp® Taq 2X Master Mix (M0287S) was from New England Biolabs (Hitchin, Hertfordshire); and DNA Gel Loading Dye (6X) (R0611), SYBR™ Safe DNA Gel Stain (S33102), and MitoTracker™ Deep Red FM (M22426) were from Thermo Scientific (Massachusetts, US). 1-Step Quantitative Reverse Transcription PCR (RT-qPCR) from RNA (1725151) was from BIO-RAD (California, US). ReverTra Ace® qPCR RT Master Mix (FSQ-201) was from Toyobo (Osaka, Japan). Detection kits for ATP, GPX, SOD, and MDA were supplied by Beyotime (Shanghai, China). Kits for NOX2 (SED308Hu) and ACACa (SEB284Hu) were derived from Youersheng (Wuhan, Hubei, China). All other reagents were from commercial sources.

### 2.2. Cell

HepG2 cells (hepatocyte cell line) were purchased from the American Type Culture Collection (ATCC, Manassas, VA). The cells were cultured in high-glucose MEM medium (Gibco) supplemented with 10% fetal bovine serum (FBS) and 1% penicillin-streptomycin at 37°C in a 95% air/5% CO_2_ cell incubator.

### 2.3. Integration of Protein-Protein Interaction Network Analysis

The STRING database (https://string-db.org/) is applied to predict possible interactions among proteins according to the function and pathway enrichment analysis.

### 2.4. DNA Fragmentation Observation

HepG2 cells were seeded in 6-well plates and treated with insulin or NaB. Total DNA was purified using the DNA extraction kit, separated by 1% agarose gel, and finally visualized using a GelDoc™ XR+ imaging system (Bio-Rad, Philadelphia, PA, USA).

### 2.5. Quantitative Real-Time PCR (Q-PCR)

Total RNA from HepG2 cells treated with insulin or NaB for 24 h were extracted using a TRIzol reagent according to the manufacturer's protocol. Concentration of RNA was determined by a NanoDrop 2000 instrument (Bio-Rad, USA). cDNA was reverse-transcripted from RNA by a cDNA synthesis kit according to the protocol from the supplier as follows: priming for 5 min at 25°C, reverse transcription for 20 min at 46°C, and RT inactivation for 1 min at 95°C. Real-time PCR was performed by FastStart Universal SYBR Green Master. Each sample was mixed with 10 *μ*l SYBR master mix, 2 *μ*l primers (mixture with both forward and reverse primers), 0.1 *μ*l cDNA, and DEPC-treated water to make up a total reaction volume of 20 *μ*l. Mixtures were circulated for 40 cycles using a high-productivity real-time quantitative PCR ViiATM7 (Life Technologies, Gaithersburg, MD, USA). The reference gene was *β*-actin. Each experiment was repeated for at least three times. Sequences for primers used in PCR analysis are listed in Table 1.

### 2.6. PCR Array Analysis

Quantitative PCR array analysis was carried out using an RT^2^ Profiler™ PCR Array Human Mitochondrial Energy Metabolism (QIAGEN). HepG2 cells were treated with high insulin or high insulin+NaB as indicated. Total RNA was extracted by TRIzol; cDNA was prepared from purified RNA using a ReverTra Ace® qPCR RT Master Mix (FSQ-201, Toyobo); the PCR array assay was analyzed by the kit using the high-productivity real-time quantitative PCR ViiATM7 (Life Technologies, Gaithersburg, MD, USA) according to the manufacturer's instruction. After data collection, relative gene expression was presented as ΔCt = Ct (GOI) − ave Ct (HKG); the fold change in the gene expression was calculated using the 2^−*ΔΔ*Ct^ method.

### 2.7. Flow Cytometry

HepG2 cells (1.5 × 10^5^ cells/well) were seeded in a 6-well plate and administrated with insulin (0.1 *μ*M) or NaB (0.5 mM) for 24 h. Cells were harvested and suspended with PBS solution. Then, the cells were stained with deep red mitochondria (50 nM), DCFH (10 *μ*M), JC-1 iodide (2.5 *μ*g/ml), or 2-NBDG (100 *μ*M) for 15 min at room temperature in the dark. The subpopulation of cells was estimated with a BD Aria III Flow Cytometer (BD Biosciences, San Jose, California, USA).

### 2.8. Knockdown of GPR43

Expressions of GPR43 in HepG2 cells were knocked down according to the protocol from the provider. In general, Lipofectamine® RNAiMAX (13778150) and GPR43 siRNA (sc-77339) were diluted in an Opti-MEM® Medium as instructed from the protocol and then were mixed at the ratio of 1 : 1. The siRNA-lipid mixture was incubated for 10 minutes at room temperature and then cocultured with the cells for 1-3 days within the cell incubator at 37°C.

### 2.9. Mitochondrial Imaging

HepG2 cells were incubated with the MitoTracker™ Deep Red staining solution (50 nM) in the dark for 20 minutes. After being washed with PBS, the mitochondria were observed under a laser confocal microscope (Leica TCS SP8, Germany).

### 2.10. Immunofluorescence Assay

Cells at the exponential state were incubated with insulin or NaB. Twenty-four hours later, cells were treated with 4% paraformaldehyde for 30 min. The cells were then blocked with 5% BSA and incubated with primary antibodies including GPR432 (1 : 200), insulin receptor (1 : 200), p-AKT (1 : 200), AKT (1 : 200), p-GSK3 (1 : 200), GSK3 (1 : 200), PGC1-*α* (1 : 200), AMPK (1 : 200), p-AMPK (1 : 200), or *β*-arrestin2 (1 : 200) at 4°C overnight. After being gently washed with PBS, cells were further incubated with FITC- or CY3-conjugated secondary antibody. The nucleus was stained with DAPI. Finally, the cells were observed under a confocal laser scanning microscope (Leica TCS SP8, Germany), and the fluorescent density was determined by ImageJ software.

### 2.11. Enzyme Immunoassay (EIA)

Levels of malondialdehyde (MDA), glutathione peroxidase (GPX), superoxide dismutase (SOD), NOX2, adenosine triphosphate (ATP), and ACACa were determined by kits according to the manufacturers' protocols.

### 2.12. Statistical Analysis

All data were obtained from more than three independent repeated experiments and were analyzed by GraphPad Prism 5 software; data that fit into the normal distribution were expressed as mean ± standard deviation (SD), and the differences among groups were analyzed by the one-way ANOVA method. Comparisons between two groups were made using Student's *t*-test. *p* < 0.05 was considered as statistically significant.

## 3. Results

### 3.1. Sodium Butyrate (NaB) Modulated Genes Related with Mitochondrial Energy Metabolism

**Figure 1:**
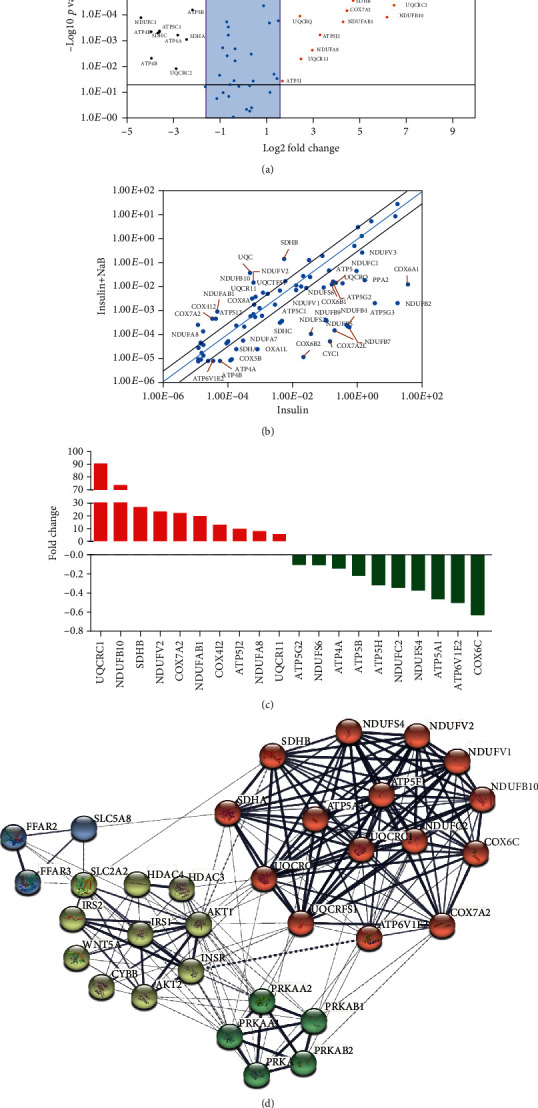
Changes in expression for mitochondrial energy metabolism-related genes between insulin resistance and insulin resistance+sodium butyrate (NaB) treatment groups. (a–c) Changes for genes related to mitochondrial energy metabolism were assayed by the RT^2^ Profiler PCR Array; (a) volcano plot for log2 fold changes in genes between groups; (b) relative expression comparison for genes between high insulin (*x*-axis) and high insulin+NaB treatment (*y*-axis) groups; (c) fold changes in gene expression for twenty representative genes after NaB administration. (d) Predicted protein-protein interaction among genes from mitochondrial energy metabolism and genes with NaB activity generated from the STRING database.

Previously, we have demonstrated that NaB promoted glycogen metabolism within hepatocytes [8] and decreased the glucose level in db/db mice [7]. As mitochondria play a pivotal role in modulating energy balance, we further carried out experiments to investigate influence of NaB on mitochondria under insulin resistance (IR) circumstances. To this end, we firstly determined changes of gene expression related with mitochondrial energy metabolism by a PCR array kit. As shown in Table 2 and Figures 1(a)–1(c), 35 genes were downregulated and 19 genes were upregulated in NaB-incubated cells compared with the model group (high insulin); among these genes, UQCRC1 was upregulated by as high as 90-folds, while COX6C was downregulated by 0.63-fold.

To predict possible mechanism and signaling pathways, protein-protein interaction among genes was generated from the STRING database. As depicted in Figure 1(d), AKT and AMPK signaling pathways play a pivotal role in modulating the top ten changed genes within the mitochondria, and receptors for short chain fatty acids (SCFAs) may influence the balance of AKT and AMPK pathways.

### 3.2. Mitochondrial Function Was Enhanced by NaB

**Figure 2:**
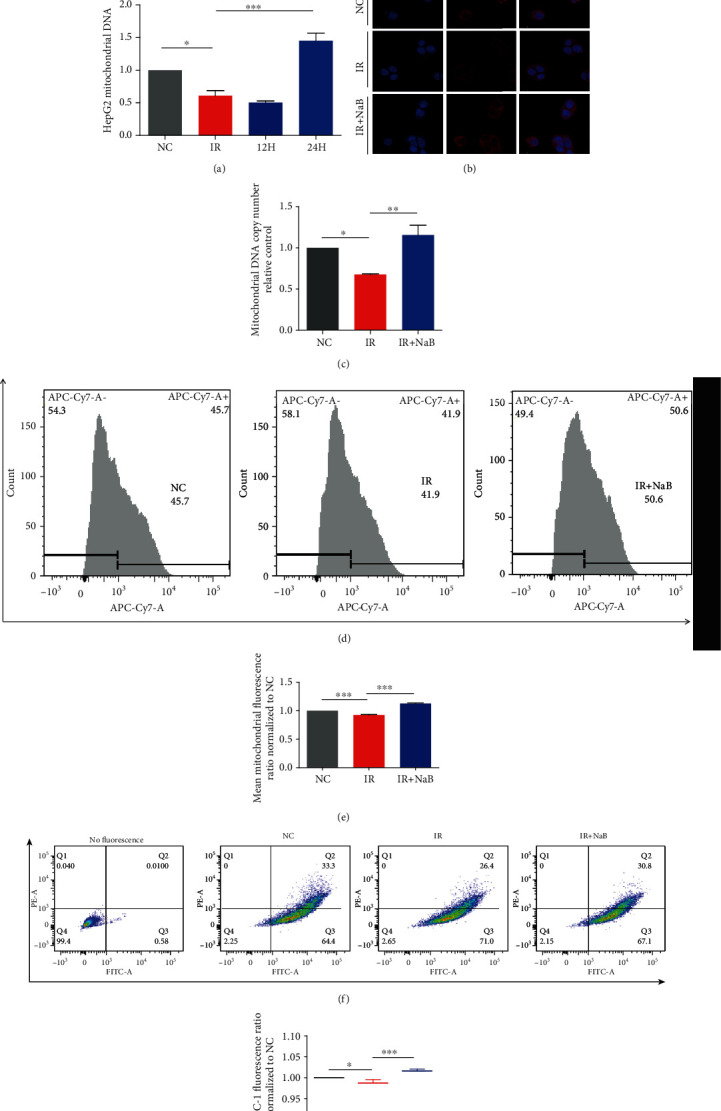
Influence of NaB treatment on function of mitochondria. (a) NaB increased content of mitochondrial DNA. (b) Mitochondria were observed by immunofluorescence method (magnification: 1200). Number of copies for mitochondrial DNA was analyzed by (c) Q-PCR and (d, e) flow cytometry. (f, g) Mitochondrial membrane potentials were determined by flow cytometry. NC: normal control; IR: high insulin-induced insulin resistance; NaB: sodium butyrate. ^∗^*p* < 0.05, ^∗∗^*p* < 0.01, and ^∗∗∗^*p* < 0.001.

To investigate role of NaB on mitochondria, we firstly determined its DNA. As shown in Figure 2(a), content of mitochondrial DNA (mtDNA) was significantly reduced by high insulin (IR), administration with NaB dramatically increased its level (*p* < 0.001 vs. IR), and the most significant effect was observed at 24 h. By immunofluorescence assay (Figure 2(b)), PCR determination (Figure 2(c)), and flow cytometry assay (Figures 2(d) and 2(e)), we confirmed that the content and copy number of mtDNA were significantly increased by NaB treatment. More importantly, mitochondrial membrane potential as probed by JC-1 was significantly elevated (Figures 2(f) and 2(g)), and ATP production was enhanced (Table 3). The above findings demonstrated that administration with NaB could significantly reverse high insulin-induced hepatocyte dysfunction by promoting the function of mitochondria.

### 3.3. NaB Ameliorated Oxidative Stress Damage under High Insulin Circumstances

**Figure 3:**
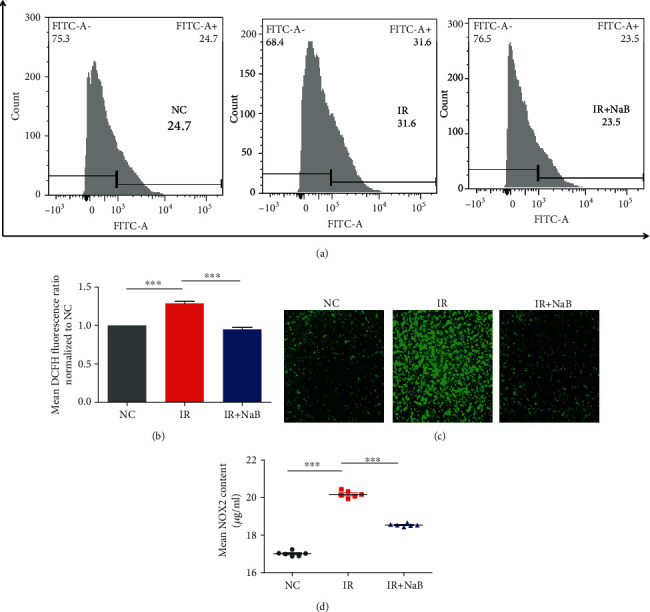
NaB ameliorated oxidative stress under high insulin settings. ROS was determined by (a, b) flow cytometer and (c) observed under a fluorescence microscope (magnification: 20). (d) Level of NOX2 was analyzed by EIA. NC: normal control; IR: high insulin-induced insulin resistance; NaB: sodium butyrate. ^∗∗∗^*p* < 0.001.

Mitochondria are the major source of reactive oxygen species (ROS), and accumulation of ROS will lead to decreased mitochondrial membrane potential and ATP production [9]. To evaluate oxidative stress after NaB administration, we determined the level of ROS by a flow cytometer (Figures 3(a) and 3(b)) and observed its content under a fluorescence microscope (Figure 3(c)); we found that insulin resistance (IR) is accompanied by overproduction of ROS, and NaB can significantly inhibit this elevation. NADPH oxidase 2 (NOX2) within mitochondria plays a pivotal role in the production of ROS. In the present study, NaB dramatically inhibited activity of NOX2 induced by IR (Figure 3(d)); other enzymes and products within hepatocytes including antioxidative SOD and GPX and prooxidative MDA were also ameliorated by NaB (Table 4).

### 3.4. NaB Mediated *β*-Oxidation of Fatty Acids and Histone Acetylation in Hepatocytes

**Figure 4:**
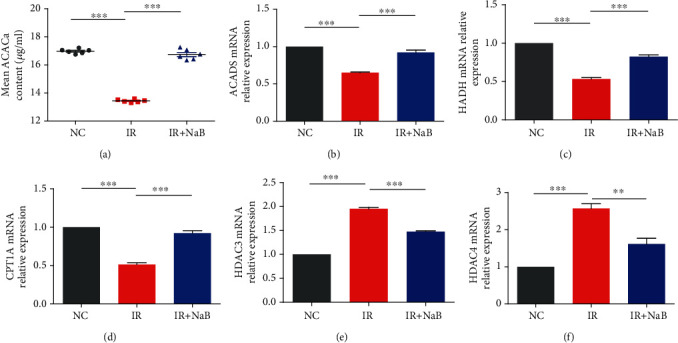
NaB modulated *β*-oxidation of fatty acids and histone acetylation. (a) Level of ACACa was determined by EIA. Expressions of (b) ACADS, (c) HADH, (d) CPT1A, (e) HDAC3, and (f) HDAC4 were analyzed by Q-PCR. NC: normal control; IR: high insulin-induced insulin resistance; NaB: sodium butyrate. ^∗∗^*p* < 0.01 and ^∗∗∗^*p* < 0.001.

Acetyl-CoA carboxylase alpha (ACACa) is the rate-limiting enzyme in fatty acid synthesis and is believed to be a novel target for endocrine disease, e.g., diabetes and obesity. In our present study, the level of ACACa was dramatically reduced by high insulin and NaB incubation significantly increased its content to the normal level (Figure 4(a)). CPT1A, HADH, and ACADS are pivotal rate-limiting enzymes in fatty acid catabolism within mitochondria during the *β*-oxidation process [10]. We found that high insulin significantly inhibited their mRNA expression, while this was reversed by NaB administration (Figures 4(b)–4(d)). In this sense, NaB application modulated the metabolism of fatty acids within hepatocytes and exhibited protective effects on the function of the mitochondrial electron transfer chain under high insulin circumstances.

Histone deacetylase (HDAC) modulates deacetylation modification of histones, thus inhibiting gene translocation and thereafter energy metabolism. Activities of rate-limiting enzymes discussed above are modulated by both the histone acetylation level and deacetylase activity. HDAC3 and HDAC4 are typical HDACs that belong to class I and II HDACs, respectively, and loss of HDAC in the liver will result in increased glycogen storage and reduced blood glucose level [11]. In the present study, we found that NaB significantly inhibited the expression of HDAC3 and 4 induced by high insulin (Figures 4(e) and 4(f)).

### 3.5. GPR43 Mediated Function of NaB on Mitochondria

**Figure 5:**
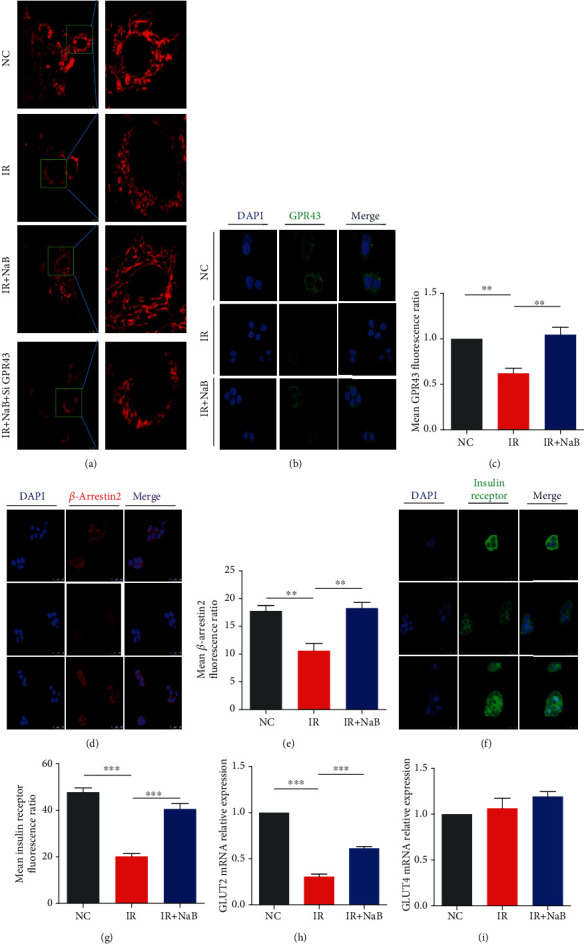
NaB increased expression of signal transduction proteins on cell membranes. (a) Shape of mitochondria within live cells were stained with MitoTracker™ Deep Red FM and observed under a confocal microscope (magnification: 1200). Expression and location for (b, c) GPR43, (d, e) beta-arrestin2, and (f, g) insulin receptor were determined by immunofluorescence and observed under a confocal microscope (magnification: 800). mRNA expression of (h) GLUT2 and (i) GLUT4 was determined by Q-PCR. NC: normal control; IR: high insulin-induced insulin resistance; NaB: sodium butyrate. ^∗∗^*p* < 0.01 and ^∗∗∗^*p* < 0.001.

Previously, we have demonstrated that GPR43 mediated the function of NaB on glycogen metabolism within the hepatocyte [8]. To explore the underlying mechanism of NaB on mitochondria, we firstly knocked down the expression of GPR43 by siRNA and observed its influence on the shape and distribution of mitochondria under a confocal microscope. As shown in Figure 5(a), high insulin (IR) induced an obvious fragmentation of mitochondria; NaB incubation significantly reversed the shape change of mitochondria via GPR43. This was further demonstrated by an immunofluorescence assay that NaB significantly increased the expression of GPR43 that was inhibited by IR (Figures 5(b) and 5(c)).

There is a previous report which indicated that *β*-arrestin2 mediated internalization of GPR43 [12], and its expression in diabetic mice was dramatically reduced. In the present study, we observed that NaB application significantly induced the expression of *β*-arrestin2 within hepatocytes (Figures 5(d) and 5(e)); more importantly, expression of the insulin receptor was also upregulated by NaB (Figures 5(f) and 5(g)). This was consistent with protein-protein interaction prediction from the STRING database (Figure 1(d)).

In the current study, we also observed mRNA upregulation of GLUT2 (Figure 5(h)) but not GLUT4 (Figure 5(i)) by NaB incubation under high insulin circumstances. This is in line with our previous findings [8], suggesting the NaB-promoted entrance of glucose into the cells may benefit energy metabolism within mitochondria.

### 3.6. AMPK-PGC1-alpha Signaling Pathways Modulated Effects of NaB on Mitochondria

**Figure 6:**
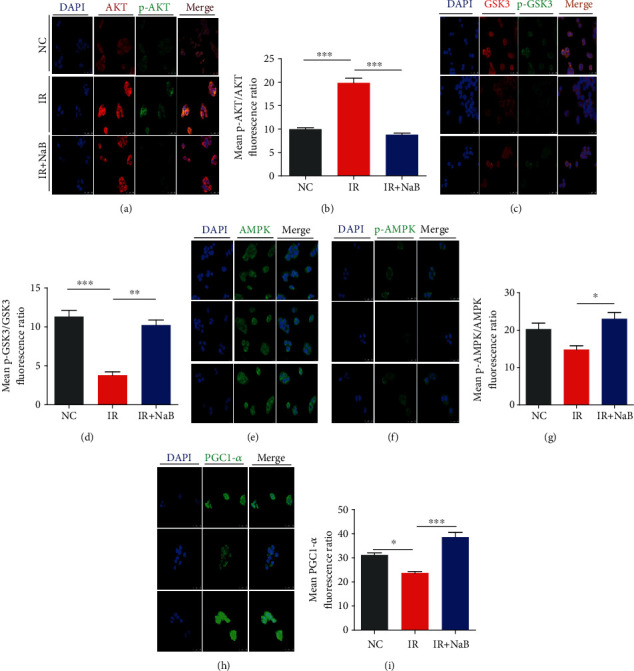
AKT and AMPK signaling pathways participated in effects of NaB on mitochondria. Expression and activation of (a, b) p-AKT/AKT, (c, d) p-GSK3/GSK3, (e–g) p-AMPK/AMPK, and (h, i) PGC1-*α* were observed by immunofluorescence method (magnification: 800). NC: normal control; IR: high insulin-induced insulin resistance; NaB: sodium butyrate. ^∗^*p* < 0.05, ^∗∗^*p* < 0.01, and ^∗∗∗^*p* < 0.001.

The AKT signaling pathway plays a pivotal role in modulating glucose uptake and metabolism within mitochondria. We found that high insulin significantly induced activation of AKT while reducing p-GSK3 compared with the normal control (*p* < 0.001), and NaB reversed this trend to the normal levels (Figures 6(a)–6(d)). On the other hand, the AMPK-PGC1-alpha signaling pathway, which modulates both biogenesis and function of mitochondria, was significantly enhanced on application of NaB (Figures 6(e)–6(i)).

## 4. Discussion and Conclusions

Insulin resistance in hepatocytes is one of the central reasons that block glucose metabolism. Recent findings have indicated the important role of cometabolism between gut microbiota and the organism. But the underlying mechanism is still not fully understood. In the current study, we demonstrated that a metabolite product from gut microbiota, sodium butyrate (NaB), can ameliorate function of hepatocytes via modulating mitochondrial metabolism.

According to a report from Kanazawa and colleagues [1], as high as 19% cases with type 2 diabetes (T2D) are accompanied with liver dysfunction. Concerning the pivotal role of the liver in mediating the metabolism of glucose and lipids, preserving its function helps to inhibit progression of T2D. With the understanding of the influence of gut microbiota towards preserving the organism in a healthy status, effects of the metabolites from microbiota against disease development have attracted more attention. It was found by Qin and colleagues that butyrate-producing bacteria were significantly reduced in a T2D population [4]. Although physiological concentration of butyrate within the liver is low, external administration with butyrate has been suggested to fight against high-fat diet-induced fatty liver [5]; this also suggested potential effects of butyrate against the development of T2D. Previously, we demonstrated in db/db mice that oral administration with NaB could significantly reduce HbA1c and diabetic inflammation [7]; more importantly, hypertrophy and steatosis of hepatocytes in db/db mice were significantly reversed by NaB, accompanied with enhancement of glycogen metabolism [8]. Our findings are in line with a report from Khan and Jena that NaB inhibited liver vascular steatosis and fat deposition [6]. But the underlying mechanism still needs to be fully explored.

Diabetes is closely related with significantly reduced mitochondrial function. In the diabetic population, mitochondrial numbers are found to be reduced [2], lipid oxidation is significantly impaired [3], and a direct relationship between mitochondria and insulin resistance is exhibited [2, 13]. To explore the relationship between NaB and liver function, we firstly carried out a PCR array assay to observe changes in gene expression. We found that NaB administration significantly increased 19 genes while downregulating as many as 35 genes in mitochondria. As most of these genes encode and regulate the composition and function of mitochondria, we further predicted protein-protein interaction between these genes and pathways related with short chain fatty acids by the STRING database. We found that there exists a possible direct relationship between short chain fatty acids and mitochondria, and the content of mitochondria and AMPK pathways is a possible reason that contributes to this relationship.

In the present study, we incubated HepG2 cell with relatively high concentration of insulin to induce insulin resistance. We found that high insulin significantly reduced both the amount and the copy of mitochondrial DNA, and mitochondrial membrane potential was decreased, while application with NaB significantly increased mitochondrial DNA and elevated the membrane potential. Our present findings suggest that NaB could increase the content of mitochondria and ameliorate its dysfunction.

Inevitable by-products of mitochondrial respiration are reactive oxygen species (ROS). In fact, mitochondria themselves contribute to the main production of ROS. Amounts of studies have demonstrated that overaccumulation of ROS and oxidative stress is one of the characteristic of diabetes. Excessive ROS in the absence of sufficient antioxidants will lead to extensive production of oxidative by-products and events, such as generation and accumulation of advanced glycation end products (AGEs), the damage of both nuclear and mitochondrial DNA (mtDNA) [14], and even cell death. Suppression of oxidative stress has been demonstrated to benefit diabetes management. SOD and GPX are two representative antioxidation enzymes. Overexpression of SOD significantly ameliorated insulin resistance in high-fat diet mice [15]. It was interesting in our present study that NaB increased both SOD and GPX expressions and decreased prooxidative NOX2, ROS, and MDA levels. This finding obviously demonstrated that NaB enhanced the function of mitochondria but did not increase the risk of oxidative stress damage.

In fact, production of ATP within mitochondria relies on oxidation. CPT1A, HADH, and ACADS are pivotal rate-limiting enzymes in fatty acid catabolism within mitochondria during *β*-oxidation [10], and their activities are modulated by histone acetylation and deacetylation. Histone deacetylase (HDAC) directly controls deacetylation modification of histones, and loss of HDAC in the liver will result in increased glycogen storage and reduced blood glucose level [11]. It has been demonstrated that HDAC protein coprecipitated with CPT1A [16]. HDAC3 and HDAC4 are typical HDACs that belong to class I and II HDACs, respectively. Reports indicated that class I HDAC contributed to mitochondrial dysfunction [17] and treatment with its inhibitor promoted energy expenditure and reduced both glucose and insulin levels by increasing PGC-1alpha activity [18]. In the present study, we observed that high insulin significantly inhibited expressions of rate-limiting enzymes of oxidation including ACACs, CPT1A, HADH, and ACADS, while their upstream HDAC was elevated, suggesting the mitochondrial electron transfer chain was blocked under high insulin settings, and NaB application ameliorated their expression. There is a previous study that demonstrated that short chain fatty acids (SCFAs), including NaB, possess a natural inhibitory effect on HDAC [19]. In this sense, NaB may modulate oxidation within mitochondria via inhibiting HDAC.

The GPR43-*β*-arrestin2 pathway has been demonstrated to mediate the function of NaB [8, 12]. GPR43 is a G protein-coupled protein on the cell membrane, and *β*-arrestin2 is one of its downstream activators that are usually recognized as a modulator of inflammation. A report has demonstrated that deficiency of *β*-arrestin2 will lead to insulin resistance [2]. A most recent study from Pydi and colleagues [21] indicated the essential role of *β*-arrestin2 in maintaining energy homeostasis within adipocytes. Another study also suggested the pivotal function of *β*-arrestin2 for maintaining euglycemia in hepatocytes [22]. But its involvement in mitochondrial dysfunction under high insulin settings is still not clear. In this study, we demonstrated that high insulin-induced GPR43 and *β*-arrestin2 reduction was significantly reversed by NaB; more importantly, both the insulin receptor and GLUT2 were upregulated on NaB administration, suggesting the amelioration of insulin resistance and energy metabolism.

Mitochondrial content is influenced by its biogenesis [23], and this process is mainly regulated by peroxisome proliferator-activated receptor coactivator-1alpha (PGC-1alpha) [24]. It was found in diabetic patients that the expression of PGC-1alpha was reduced [25], and upregulation of PGC-1alpha can significantly increase both insulin sensitivity and lipid oxidation [26]. Studies have demonstrated that phosphorylation of AMPK will activate PGC-1alpha, increase expression of mitochondria-related genes [27], and promote mitochondrial biogenesis, while HDAC1 and HDAC3 have been found to repress the transcriptional activity of PGC-1alpha [28]^–^ [30]. A recent report from Yoshida and colleagues demonstrated that knockdown of GPR43 will reduce SCFA-induced activation of AMPK [31]. Our findings in this study obviously suggest that NaB promoted the biogenesis of mitochondria via promoting AMPK-PGC1-alpha and blocking the HDAC signaling pathway.

**Figure 7:**
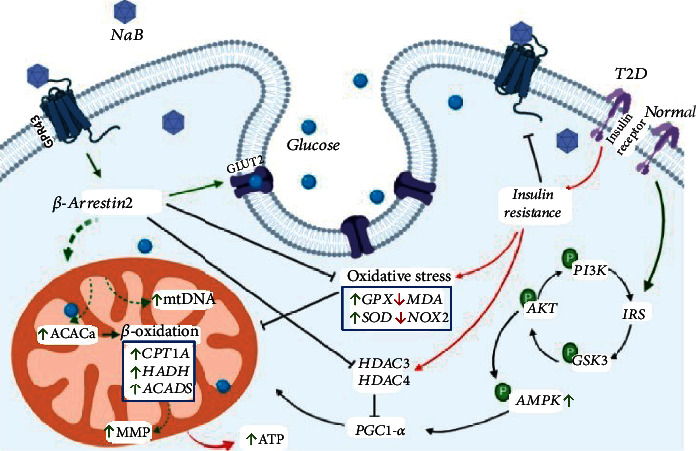
Mechanism of sodium butyrate (NaB) on modulating high insulin-induced dysfunction of mitochondria within hepatocytes. T2D: type 2 diabetes; MMP: mitochondrial membrane potential.

In conclusion, we found in our present study that sodium butyrate administration could significantly promote biogenesis and function of mitochondria under high insulin circumstances, and the GPR43-*β*-arrestin2-AMPK-PGC1-alpha signaling pathway contributed to these effects (Figure 7). Our present findings obviously provide new insight on the pivotal role of metabolites from microbiota in maintaining euglycemia.

## Figures and Tables

**Table 1:** List of sequences for primers used in PCR analysis.

	Forward primers (5′ to 3′)	Reverse primers (5′ to 3′)
GLUT2	GACAGTGAAAACCAGGGTCC	TGTGCCACACTCACACAAGA
GLUT4	GCCCTAACTTTCTTCCTCTCCCT	CCGACCTTTGGTTTCTTCTCTCA
HDAC3	CTGTGTAACGCGAGCAGAAC	GCAAGGCTTCACCAAGAGTC
HDAC4	CTGGTCTCGGCCAGAAAGT	CGTGGAAATTTTGAGCCATT
ACADS	CCCATCTTCTTCACCTGAGC	ACACACCAGATGTTGCTCCA
HADH	ACCCTGAGCACCATAGCGA	CAGCGAATCGGTCTTGTCTGG
CPT1A	ATCAATCGGACTCTGGAAACGG	TCAGGGAGTAGCGCATGGT
*β*-Actin	GTTGTCGACGACGAGCG	GCACAGAGCCTCGCCTT
nDNA	TGAGGCCAAATATCATTCTGAGGGGC	TTTCATCATGCGGAGATGTTGGATGG
mtDNA	ACATGATTAGCAAAAGGGCCTAGCTTGGACTCAGA	TGCACCTGCTCTGTGATTATGACTATCCCACAGTC
MinArc	CTAAATAGCCCACACGTTCCC	AGAGCTCCCGTGAGTGGTTA
*β*2M	GCTGGGTAGCTCTAAACAATGTATTCA	CCATGTACTAACAAATGTCTAAAATGGT

**Table 2:** Expression of mitochondrial energy metabolism-related genes between insulin resistance (insulin) and insulin resistance+sodium butyrate (NaB/insulin) treatment groups.

Gene	Accession no.	Normalized ratio (insulin+NaB/insulin)	*p* value	Up/downregulation
ATP12A	NM_001185085.1	1.2014	0.0531	—
ATP4A	NM_000704.3	0.1417	0.0006	Down
ATP4B	NM_000705.4	0.0654	0.0004	Down
ATP5A1	NM_001001935.3	0.4648	0.1675	Down
ATP5B	NM_001686.4	0.2171	0.0001	Down
ATP5C1	NM_001001973.3	0.0827	0.0004	Down
ATP5F1	NM_001688.5	0.6247	0.0002	—
ATP5G1	NM_005175.3	1.1209	0.4700	—
ATP5G2	NM_001330269.1	0.1045	<0.0001	Down
ATP5G3	NM_001190329.2	0.0006	<0.0001	Down
ATP5H	NM_006356.3	0.3197	0.0593	Down
ATP5I	NM_007100.4	0.0655	<0.0001	Down
ATP5J	NM_001003703.1	3.1998	0.0354	Up
ATP5J2	NM_004889.5	9.8811	0.0006	Up
ATP5L	NM_006476.5	0.5051	<0.0001	—
ATP5O	NM_001697.3	0.6247	0.0002	—
ATP6V0A2	NM_012463.4	0.6408	0.0006	—
ATP6V0D2	NM_152565.1	0.6247	0.0002	—
ATP6V1C2	NM_001039362.2	2.5339	0.0187	Up
ATP6V1E2	NM_001318063.2	0.5028	0.0212	Down
ATP6V1G3	NM_001320218.1	1.2351	0.5168	—
BCS1L	NM_001079866.2	0.7551	0.8782	—
COX4I1	NM_001861.6	0.6543	0.1425	—
COX4I2	NM_032609.3	13.1592	<0.0001	Up
COX5A	NM_004255.4	0.7621	0.0348	—
COX5B	NM_001862.3	1.3146	0.3899	—
COX6A1	NM_004373.4	0.0004	<0.0001	Down
COX6A2	NM_005205.4	2.7473	0.0264	Up
COX6B1	NM_001863.5	0.0837	<0.0001	Down
COX6B2	NM_001369798.1	0.0006	<0.0001	Down
COX6C	NM_004374.4	0.6320	0.0003	Down
COX7A2	NM_001865.4	22.0800	0.0001	Up
COX7A2L	NM_004718.4	0.0008	<0.0001	Down
COX7B	NM_001866.3	0.7817	0.0019	—
COX8A	NM_004074.3	5.4945	0.0001	Up
COX8C	NM_182971.3	3.6587	<0.0001	Up
CYC1	NM_001916.5	0.0004	<0.0001	Down
LHPP	NM_022126.4	0.6247	0.0002	—
NDUFA1	NM_004541.4	1.3579	0.0354	—
NDUFA10	NM_001322019.1	0.6247	0.0002	—
NDUFA11	NM_001193375.2	0.7745	0.0059	—
NDUFA2	NM_002488.5	2.2212	0.0970	—
NDUFA3	NM_004542.4	1.2757	0.0033	—
NDUFA4	NM_002489.4	2.8706	<0.0001	—
NDUFA5	NM_001291304.1	0.6247	0.0002	—
NDUFA6	NM_002490.6	2.8050	0.0002	Up
NDUFA7	NM_005001.5	0.6758	0.0504	—
NDUFA8	NM_001318195.2	7.9521	0.0023	Up
NDUFAB1	NM_005003.3	19.8537	0.0002	Up
NDUFB10	NM_004548.3	73.5847	0.0001	Up
NDUFB2	NM_004546.3	0.0001	0.0001	Down
NDUFB3	NM_001257102.2	0.0657	0.0044	Down
NDUFB4	NM_001168331.2	0.0005	0.0001	Down
NDUFB5	NM_002492.4	0.0008	<0.0001	Down
NDUFB6	NM_002493.5	0.8693	0.0576	—
NDUFB7	NM_004146.6	0.0003	<0.0001	Down
NDUFB8	NM_005004.4	0.5351	0.1035	—
NDUFB9	NM_005005.3	0.0035	0.0001	Down
NDUFC1	NM_001184986.1	0.0473	0.0001	Down
NDUFC2	NM_004549.6	0.3450	<0.0001	Down
NDUFS1	NM_005006.7	0.6176	0.0039	—
NDUFS2	NM_004550.4	0.0031	<0.0001	Down
NDUFS3	NM_004551.3	0.6247	0.0002	—
NDUFS4	NM_002495.4	0.3749	<0.0001	Down
NDUFS5	NM_004552.3	0.6247	0.0002	—
NDUFS6	NM_004553.6	0.1052	<0.0001	Down
NDUFS7	NM_024407.5	2.1856	0.0002	Up
NDUFS8	NM_002496.4	0.6247	0.0002	—
NDUFV1	NM_007103.4	0.0424	<0.0001	Down
NDUFV2	NM_021074.5	23.3389	<0.0001	Up
NDUFV3	NM_021075.4	0.1932	<0.0001	Down
OXA1L	NM_005015.5	0.0301	0.0001	Down
PPA1	NM_021129.4	0.5502	0.0019	—
PPA2	NM_176869.3	0.0111	<0.0001	Down
SDHA	NM_004168.4	0.1832	0.0008	Down
SDHB	NM_003000.3	26.9336	<0.0001	Up
SDHC	NM_003001.5	0.0785	0.0005	Down
SDHD	NM_003002.4	2.8905	<0.0001	Up
UQCR11	NM_006830.4	5.6752	0.0049	Up
UQCRC1	NM_003365.3	90.3843	<0.0001	Up
UQCRC2	NM_003366.4	0.1332	0.0119	Down
UQCRFS1	NM_006003.3	4.9178	<0.0001	Up
UQCRH	NM_006004.4	1.8506	<0.0001	—
UQCRQ	NM_014402.5	0.0390	<0.0001	Down

**Table 3:** ATP production among groups (*n* = 3).

Group	ATP (nM/mg prot)
NC	349.39 ± 38.32
IR	250.24 ± 3.13^∗∗^
IR+NaB	333.15 ± 51.77^#^

NC: normal control; IR: high insulin-induced insulin resistance; NaB: sodium butyrate. ^∗∗^*p* < 0.01 vs. NC; ^#^*p* < 0.05 vs. IR.

**Table 4:** Activity of enzymes participating in oxidative stress among groups (*n* = 3).

Group	SOD (mU/mg prot)	GPX (mU/mg prot)	MDA (nM/mg prot)
NC	222.80 ± 1.23	59.32 ± 4.11	1.93 ± 0.14
IR	184.12 ± 8.99^∗∗^	51.88 ± 2.25^∗∗^	3.35 ± 0.1^∗∗^
IR+NaB	208.10 ± 16.87^##^	68.86 ± 9.02^##^	2.24 ± 0.04^##^

NC: normal control; IR: high insulin-induced insulin resistance; NaB: sodium butyrate. ^∗∗^*p* < 0.01 vs. NC; ^##^*p* < 0.01 vs. IR.

## Data Availability

Data in this paper are available on PubMed or Scopus. Any previous paper not accessible could be requested from the corresponding author.
